# Application of family therapy in a case of anorexia nervosa

**DOI:** 10.1192/j.eurpsy.2023.1797

**Published:** 2023-07-19

**Authors:** C. Díaz Mayoral, I. Romero Gerechter, E. Arroyo Sánchez, M. Martín Velasco, A. Sanz Giancola, M. Martín de Argila

**Affiliations:** 1 Psychiatry, Hospital Universitario Príncipe de Asturias; 2 Psychiatry, Hospital Dr. Rodriguez Lafora, Madrid, Spain

## Abstract

**Introduction:**

Anorexia nervosa is a behavioral mental disorder, characterized by body dysmorphia, an intense fear of gaining weight and behaviors that interfere with this, in addition to a restriction of food intake, associated usually with medical complications, even a considerable risk of death.

Several psychotherapeutic approaches have been used along last decades. Until relatively recently, parents have been recognized as part of the problem, but nowadays we involve them into the therapeutic process through family therapy based on a systemic approach, recommended in current published clinical guidelines and research findings, with consistent evidence, as the first-line treatment of patients with anorexia nervosa.

**Objectives:**

A case of a patient with anorexia nervosa, is presented followed by a theoretical review on the topic.

**Methods:**

A case is presented with a bibliographic review.

**Results:**

A 24-yeard-old female was hospitalized for renutrition due to a significant weight loss and multiple physical symptoms. After 4 months without progress, the patient was transferred to the psychiatric ward.

Once there, physical stabilisation was achieved with family therapy and pharmacological treatment, based on progressive administration of Clomipramine, previously assessed by Cardiology, which improved rumination and obsessive behaviour. We conduct daily individual and weekly family interviews, working on family dynamics, emotional regulation strategies and more adaptive ways of communication. Likewise, several lines of action were found in the systemic work: peripheral father; maternal over-involvement; fraternal rivalry; difficulties of interaction between all of them, derived from “the role of the sick person” and intra-family communication around the illness. Finally, showed effectiveness in terms of an improvement in interpersonal relationships, greater assertiveness and an optimistic attitude with an active search for coping strategies.

**Conclusions:**

Historically, parents have been recognized by a causal factor in the pathogenesis of this disorder. Nevertheless, the abolition of the emphasis on family responsibility, motivated by a philosophic and evidence-based, has allowed us to see them as an essential resource in aiding the patient in the improvement process. This parental involvement has resulted in a relevant reduction in morbidity, as well as a significant decrease treatment attrition rates. It has been noted a re-establishment in other individual and family factors such as self-esteem, quality of life, and some aspects of the experiences of caregiving, and behavioral symptoms have been resolved.

**Disclosure of Interest:**

None Declared

